# COVID-19 and cancer registries: learning from the first peak of the SARS-CoV-2 pandemic

**DOI:** 10.1038/s41416-021-01324-x

**Published:** 2021-03-25

**Authors:** Alvin J. X. Lee, Karin Purshouse

**Affiliations:** 1 UCL Cancer Institute, University College London, London, UK; 2 CRUK Edinburgh Cancer Research Centre, The University of Edinburgh, Edinburgh, UK

**Keywords:** Cancer, Viral infection

## Abstract

The SARS-Cov-2 pandemic in 2020 has caused oncology teams around the world to adapt their practice in the aim of protecting patients. Early evidence from China indicated that patients with cancer, and particularly those who had recently received chemotherapy or surgery, were at increased risk of adverse outcomes following SARS-Cov-2 infection. Many registries of cancer patients infected with SARS-Cov-2 emerged during the first wave. We collate the evidence from these national and international studies and focus on the risk factors for patients with solid cancers and the contribution of systemic anti-cancer treatments (SACT—chemotherapy, immunotherapy, targeted and hormone therapy) to outcomes following SARS-Cov-2 infection. Patients with cancer infected with SARS-Cov-2 have a higher probability of death compared with patients without cancer. Common risk factors for mortality following COVID-19 include age, male sex, smoking history, number of comorbidities and poor performance status. Oncological features that may predict for worse outcomes include tumour stage, disease trajectory and lung cancer. Most studies did not identify an association between SACT and adverse outcomes. Recent data suggest that the timing of receipt of SACT may be associated with risk of mortality. Ongoing recruitment to these registries will enable us to provide evidence-based care.

## Background

Since the SARS-Cov-2 pandemic began at the start of 2020, cancer teams around the world have adapted their practice in the aim of protecting patients. After a period where restrictions were eased, many countries have returned to national lockdowns as case numbers rise. The challenge of protecting patients with cancer in a world where SARS-Cov-2 is endemic has become all the more apparent. During the first international phase of the SARS-Cov-2 pandemic, cancer services were paused as it was feared that patients with cancer were at particular risk of severe infection. The immunomodulatory effect of both cancer and many systemic anti-cancer treatments (SACT) was identified as a risk factor for this group, particularly given the evidence from previous infection outbreaks.^1–3^ This was supported by early evidence from Wuhan province, China, that cancer patients, and particularly those who had recently received chemotherapy or surgery, were at increased risk.^4,5^ Cancer treatment (including SACT and radiotherapy) and care inherently requires physical contact and can result in side effects, thereby further increasing the risk to patients. Many countries adopted ‘shielding’ policies, advising patients to stay at home.^6^ Remote consultations and rationalisation of treatment modalities were introduced to minimise the risks for patients requiring active treatment. Cancer services were also reorganised to allow clinical services to prioritise the high clinical acuity of COVID-19 patients, including redeploying staff to other clinical areas. Overall, cancer teams and their patients made huge adaptations in the face of significant uncertainty.^3,7,8^

As further waves of SARS-Cov-2 take hold, cancer teams around the world must make decisions about how to move forward. There is significant concern that delays to both cancer diagnosis and treatment will lead to the mortality from cancer exceeding that from SARS-Cov-2.^7,9,10^ Further, the backlog of patients for whom diagnosis and treatment were delayed during the first wave will see an increasing need for all forms of cancer treatment, and likely at a more advanced cancer stage. Understanding which treatment modalities confer the highest risk is vital in order to discuss the relative risks with patients and facilitate collaborative decision-making.

**Fig. 1:**
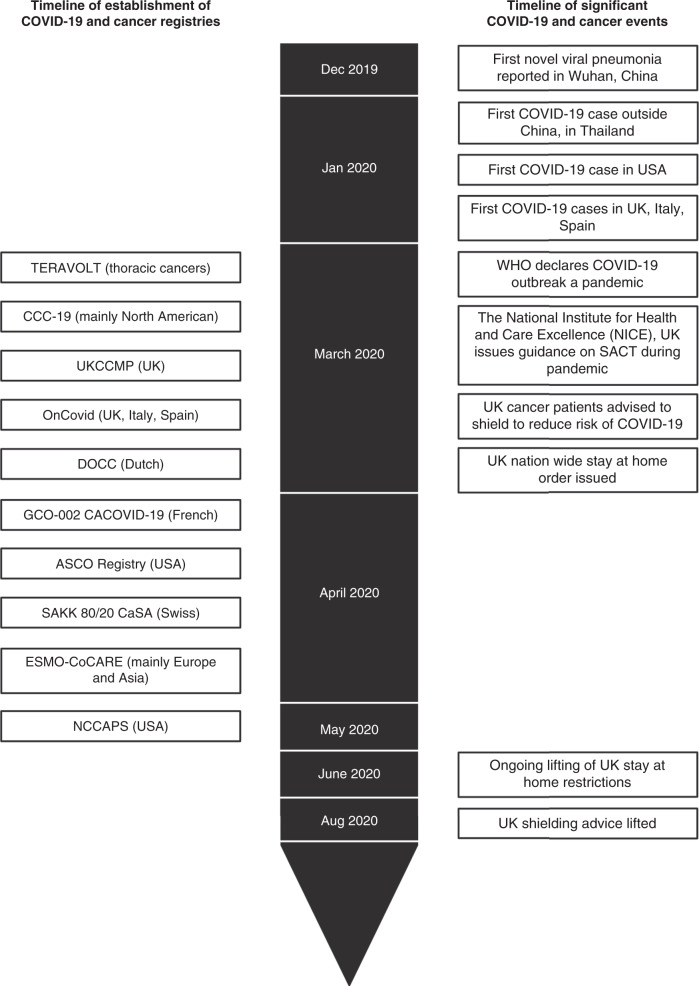
Timeline of establishment of COVID-19 and cancer registries, and significant COVID-19 and cancer events. Figure created with BioRender.com.

Cancer registries of patients infected with SARS-Cov-2 emerged during the first wave to address these uncertainties, and to date they likely offer the most comprehensive clinical data to guide cancer teams. These have ranged from local or regional databases to national and international registries. In this review, we aim to collate the evidence from large national and international registries and highlight trends and challenges these data present. Fig. 1 illustrates the timeline of establishment of major COVID-19 and cancer registries, and significant COVID-19 and cancer events. Overall, these studies suggest that patients with cancer who develop SARS-Cov-2 have a higher probability of death compared with patients without cancer. Common risk factors for mortality following SARS-Cov-2 infection identified in patients with cancer include age, male sex, smoking history, the number of comorbidities and poor performance status (PS). Oncological features that may predict for worse outcomes include tumour stage and progressive disease, and possibly lung cancer. Evidence for the safety of SACT is more conflicting with most studies identifying no association with adverse outcomes, while others report that in some groups, chemotherapy or immunotherapy may confer an increased risk. Recent data have suggested that timing of receipt of SACT in relation to SARS-Cov-2 diagnosis may affect outcomes following SARS-Cov-2 infection.^11^ A key challenge underlying any comparisons lie in the diversity between these registries and the populations they describe, noting that the population of cancer patients actively treated during the first wave of SARS-Cov-2 may not reflect the overall cancer population.

## Methods

A search of the PubMed database, and European Society for Medical Oncology (ESMO) and American Society of Clinical Oncology (ASCO) meeting abstracts was undertaken for articles up to October 14, 2020. Keywords used were “COVID-19”, “SARS-Cov-2”, “cancer”, “malignancy”. Registries that were described as national or multinational were included. We note that other registries have been established and have not yet published their findings, such as the ESMO-CoCARE Registry^12^ and NCI COVID-19 in Cancer Patients Study (NCCAPS)^13^ and these studies have been excluded from the review. Table 1 summarises the published output from the registries identified, which are explored in more detail below.

**Table 1:** National and international COVID-19 and cancer registries.

Study	No. of patients	Population	Study dates	Definition of cancer	Definition of COVID-19	Mortality & risk factors significant in multivariate analysis	Effect of recent anti-cancer therapy
**China Nationwide Analysis**^4^	18	Solid organ and haematological cancers; China	Up to 31 January 2020	Cancer history (0–16 years of follow-up) four patients (28.6%) active anti-cancer treatment (<30 days)	Laboratory	39% risk of severe events. Older age	Chemotherapy or surgery associated with worse outcomes, OR 5.34 (95% CI 1.80–16.18; *P* = 0.0026)
**ISARIC-CCP Cancer UK**^18^	1797	“Malignant neoplasm”. Hospitalised patients in the UK with COVID-19 (including non-cancer)	17 January to 17 August 2020	“Malignant neoplasm” 24% on “active treatment” (not defined)	Laboratory or high likelihood of COVID-19	40.5% mortality in cancer patients vs 28.5% in those without cancer 1.62 (95% CI 1.56–1.68, *P* < 0.001)	No effect detected in those <50 years Increased risk of mortality in those aged 50–69 years, HR 8.54 (95% CI 7.01–10.4; *P* < 0.001), aged 70–79 years HR 9.55 (95% CI 8.09–11.26; *P* < 0.001) and aged >80 years, HR 11.64 (95% CI 9.94–13.63; *P* < 0.001)
**TERAVOLT**^9^	200	Thoracic cancers; USA, Spain, Switzerland, Italy, France, Netherlands, UK, China	26 March to 12 April 2020 Median follow-up 15 days	Thoracic cancer, timeframe not defined. 74% on active cancer treatment.	Laboratory, radiological or clinical	33% Smoking history	No effect detected
TERAVOLT^21^	1012	Thoracic cancers; 20 countries	Up to 15 July 2020	Thoracic cancer, timeframe not defined 65% on active treatment	See above	32% Age, smoking, cancer stage, steroid use	No treatment or chemotherapy was found to increase risk of death compared with immunotherapy, chemotherapy-immunotherapy, or targeted treatments. OR 1.4 (95% CI 1.02–2.0; *P* = 0.03)
**CCC-19**^14^	928	Solid organ and haematological cancers; USA, Spain, Canada	March 17, 2020 to April 16, 2020. Follow-up until May 7, 2020 Median follow-up 21 days	Active and previous cancer 39% on active cancer treatment (within < 4 weeks)	Laboratory or radiological	13% Age, male, smoking, increasing number of comorbidities, ECOG PS > 2, active cancer	No effect detected
CCC-19^11,15^	3920	See above	Up to July 31, 2020	Active and previous cancer 42% received systemic anti-cancer treatment (SACT) within 12 months	See above	14% overall, 23% in hospitalised patients Age, male, smoking, black vs white ethnicity, ≥3 number of comorbidities, ECOG PS ≥ 2, progressive cancer, treatment within 3 months, haematological malignancies, multiple malignancies	Chemotherapy within 2 weeks, standardised mortality rate (SMR) 1.30 (95% CI 1.00–1.67). Chemotherapy-immunotherapy within 2 weeks, SMR 2.13 (95% CI 1.02–3.91). Targeted treatments (including anti-CD20) within 1–3, SMR 2.31 (95% CI 1.59–3.25). Within 3–12 months SMR 1.91 (95% CI 1.02–3.27)
**UKCCMP**^22^	800	Solid organ and haematological cancers; UK	March 18, 2020 to April 26, 2020	Cancer treated within <1 year or metastatic disease 76% were on active cancer treatment (<4 weeks)	Laboratory	28% Age, male, comorbidities (hypertension & cardiovascular disease)	No effect detected
UKCCMP^16^	1044 (817 non-haematological malignancies)	Solid organ and haematological cancers; UK Compared with UK cancer population without COVID-19	March 18, 2020 to May 8, 2020 Median follow-up 6 days	See above 56% of solid tumour patients were on active cancer treatment (<4 weeks)	See above	30.6% Age, haematological malignancies	Increased risk in haematological patients receiving chemotherapy <4 weeks (OR 2.09, 95% CI 1.09–4.08)
**OnCovid**^38^	204	Solid organ and haematological cancers; UK, Spain, Italy	February 26, 2020 to April 1, 2020	Active and previous cancer 50% were on SACT (< 4 weeks)	Laboratory	29% Age, more than two comorbidities	No effect detected
OnCovid^19^	890	Solid organ and haematological cancers; UK, Spain, Italy, Germany	February 26, 2020 to April 1, 2020 Mean follow-up 19 days	See above 53.8% were on SACT (< 4 weeks)	See above	33.6% Age, male, more than 2 comorbidities	No deleterious effect detected (nb: SACT was associated with better outcomes (HR 0.71, 95% CI 0.53–0.95; *P* = 0.019) but cohort younger, less comorbid and more female patients)
**Dutch Oncology COVID-19 Consortium (DOCC)**^23^	351	Solid organ and haematological cancers; Netherlands	March 27, 2020 to May 4, 2020	Active and previous cancer diagnosed <5 years or ongoing impact 47% active anti-cancer treatment (<30 days)	Laboratory or radiological	32.3% Male, age, prior or other cancer, haematological or lung cancer	No effect detected
**GCO-002 CACOVID-19**^25^	1289	Solid organ; France	March 1, 2020 to June 11, 2020 Median follow-up 34 days	Histologically confirmed solid tumour 38% on SACT within 4 weeks, 59% on SACT within last 3 months	Laboratory, radiological or symptoms	29% Male, performance status, updated Charlson comorbidity index (uCCI), admission to ICU	No effect detected overall, although note increased risk of death (OR 1.53, 95% CI 1.0–2.34; *P* = 0.05) in patients with PCR-confirmed COVID-19 who had received cytotoxic chemotherapy in the 4 weeks or 3 months prior to COVID-19
**SAKK 80/20 CaSA**^24^	359	Solid organ and haematological cancers; Switzerland	March 1, 2020 to July 16, 2020	51.8% active anti-cancer treatment (<30 days)	Laboratory or radiological	17.8% Age, ICU admission, non-curative treatment	No effect detected

*CI* confidence interval, *HR* hazard ratio, *OR* odds ratio.

## Covid-19 and cancer registries

### CCC-19

The Clinical impact of COVID-19 on patients with Cancer (CCC-19) cohort study included adult patients with active or previous cancer with a serological confirmation of SARS-Cov-2 infection in patients from over 120 institutions across the USA, Canada and Spain. The first analysis of 928 patients showed 39% were on active anti-cancer treatment, and only 43% had active (measurable) cancer. The mortality rate was 13%, and after logistic regression analysis, age, male gender, smoking, comorbidities >2, active cancer and ECOG PS > 2 were associated with increased 30-day mortality. Neither cancer type nor recent anti-cancer therapy or surgery were associated with increased mortality.^14^ This is the lowest mortality rate seen in any cancer registry study and perhaps reflects a higher SARS-Cov-2 testing strategy, compared with many European countries where testing was initially only on a symptomatic basis.

The most recent analysis from CCC-19 of 4169 patients identified male gender, age, smoking, multiple comorbidities (>2), ECOG PS > 0, progressive cancer, haematological or other concurrent solid-organ cancer and severe presenting illness with COVID-19 with worse outcomes.^15^ A simultaneous analysis of 3920 patients identified the highest mortality was seen in patients who had received treatment in the previous 1–3 months. Standardised incidence ratios were highest in those who had received chemo- or chemoimmunotherapy within 2 weeks of SARS-Cov-2 infection. It is notable that haematology patients made up 26% of this cohort, given the finding from the UK registry of a higher mortality rate in this group.^11,16^ This is particularly as an increased mortality rate in targeted treatments appears to be driven by anti-CD20 agents such as rituximab which are used almost exclusively in haematological malignancies.

### ISARIC—clinical characterisation protocol (CCP)-cancer UK

The International Severe Acute Respiratory and Emerging Infections Consortium (ISARIC)-4C COVID-19 Clinical Information Network (CO-CIN) is a UK-wide collaborative collecting data on hospital in-patients with confirmed or clinically likely SARS-Cov-2 infection. Data on 20,133 patients hospitalised due to SARS-Cov-2 suggested cancer was a factor associated with mortality.^17^ In their first cancer-specific analysis presented at the ESMO 2020 congress, 1797 (8.6%) of participants had malignant neoplasm, with 35% discharged alive, and a mortality rate of 35%. With updated data presented at ESMO, cancer was identified as a risk factor for death in all age groups, with a mortality rate of 40.5%.^18^ It is notable that the definition of cancer does not describe whether cancers are historical or active, solid organ or haematological, or the cancer stage or status of cancer treatment. All patients in this cohort were hospitalised patients and therefore likely had a more severe SARS-Cov-2 disease than other registries.

### OnCOVID

The OnCovid study reported on 890 patients from the UK, Spain, Italy and Germany. They included patients with current and previous cancer – 62.5% were defined as having ‘active malignancy’.^19^ Just over half (53.8%) of the whole cohort had received SACT in the previous 4 weeks. They reported a mortality rate of 33.6%, comparable with those from other European patient cohorts, and similarly identified age, male gender and more than two comorbidities as risk factors. Interestingly, while they also reported no negative survival impact of SACT overall, they also identified that SACT was associated with better outcomes (HR 0.71,  95% CI 0.53–0.95; *P* = 0.019) but noted that this cohort had a greater proportion of young, female and less comorbid patients.

### TERAVOLT

This cohort is composed solely of thoracic cancers, including small and non-small cell lung cancers and rarer subtypes such as pulmonary neuroendocrine neoplasms. In their preliminary findings, they identified that 76% of patients were hospitalised and 33% died. Multivariate analysis only identified smoking history as a risk factor for death.^20^ An update presented at the ESMO 2020 congress of 1012 patients from 20 countries identified age (>65), cancer stage (stage > III), current smoker status and steroids prior to SARS-Cov-2 infection confirmation as risk factors for death after multivariate analysis. Neither chemotherapy nor tyrosine kinase inhibitors (TKIs) were associated with increased mortality, and this analysis identified a reduced risk for mortality for patients on immunotherapy.^21^ In the TERAVOLT cohort, the majority of patients were on active cancer treatment (74% in the first analysis, 65% in the second), the highest of all the SARS-Cov-2 cancer registries. Despite this, the mortality rate was not notably higher.

### UKCCMP

This UK-wide registry included patients from over 70 centres across the UK and reported on 800 patients with cancer treated within the last 12 months or metastatic cancer. The overall mortality rate was 28% in a population of symptomatic patients seen in secondary care. This early analysis highlighted a similar proportion of patients on active treatment as TERAVOLT (76% in the last 4 weeks prior to SARS-Cov-2 confirmation) and identified age, male gender and comorbidities as risk factors for mortality. Similar to other registries reporting at this time, recent chemotherapy was not associated with a worse mortality rate after multivariate analysis.^22^ A subsequent analysis compared the UKCCMP cohort with a non-SARS-Cov-2 cancer cohort via the UK Office of National Statistics (ONS). A relative over-representation of haematological malignancies in the UKCCMP cohort suggested this group may be more vulnerable to SARS-Cov-2 infection. Further multivariate analysis highlighted a higher mortality rate with recent chemotherapy in this group.^16^

### Dutch oncology COVID-19 consortium (DOCC)

This study of solid organ and haematological malignancies evaluated 351 patients with serological or radiological evidence of SARS-Cov-2 infection. The overall mortality rate was 32.3%, with age >65 years, male gender, previous/other cancer, and active haematological or lung cancer identified as risk factors of death after multivariate analysis. Recent treatment (<30 days) had no bearing on outcome.^23^

### SAKK 80/20 CaSA

A registry of 357 patients from 23 Swiss centres was presented at the ESMO 2020 congress. 57% of patients had received anti-cancer treatment within 3 months of their diagnosis of SARS-Cov-2. They observed a mortality rate of 18%, and identified age and treatment with palliative intent as being associated with worse outcomes—full analysis is awaited.^24^

### GCO-002 CACOVID-19

In this French nationwide multicentre study of cancer and SARS-Cov-2, patients were included if they had cancer and excluded for those treated curatively more than 5 years ago with no evidence of recurrent disease.^25^ Analysis of 1289 patients again highlighted male gender and PS > 2 as being associated with death, in addition to the updated Charlson comorbidity index (uCCI) and admission to ICU. A thoracic primary tumour and corticosteroids prior to SARS-Cov-2 diagnosis were associated with increased SARS-Cov-2 severity, defined as admission to ICU and/or mechanical ventilation and/or death. Multivariate analysis suggested no association between cytotoxic chemotherapy within 3 months (OR 1.32,  95% CI 0.92–1.89; *P* = 0.13), however this became significant when only PCR-confirmed SARS-Cov-2 patients (*n* = 952) were considered (OR 1.53, 95% CI 1.00–2.34; *P* = 0.05). Given that this result is at the limit of significance, this result should be considered alongside the other studies described here which more comprehensively contrast with this finding. This could be a result of reporting bias, which the authors themselves propose in their discussion.^25^

## Discussion

### Do patients with cancer do worse?

Overall, there is a growing body of evidence that patients with a history of cancer appear to have a higher mortality rate compared with those without cancer. These large national and international studies demonstrate that the mortality rate of patients with cancer who are infected with SARS-Cov-2 ranges between 13 and 40.5%.

Most of these studies did not have a direct comparator arm with patients without cancer. However, ISARIC shows that within the cohort, patients with cancer had poorer outcomes with a 40.5% risk of mortality in those with cancer compared with 28.5% in those without cancer (HR 1.62, 95% CI 1.56–1.68; *P* < 0.001).^17,18^ Additionally, there have been several published studies, the majority of them within single healthcare systems from Europe, China and the USA, that have also demonstrated that a history of solid tumours is an independent risk factor for mortality when compared with patients without cancer.^26–31^ Only one, a European analysis of SARS-Cov-2-infected patients with (*n* = 435) and without (*n* = 2636) cancer, showed no relationship between cancer and SARS-Cov-2 outcome once age, gender and comorbidities were accounted for.^32^ They did note that patients with cancer were more likely to be older and with pre-existing conditions. This outlier result may be due to differences in the way in which cases were identified. The weight of evidence favours cancer as an independent risk factor, noting that cancer is itself associated with other SARS-Cov-2-related risk factors such as increasing age and certain comorbidities.

Cancer, even within solid cancers, comprises a collection of very heterogeneous diseases with different outlooks and treatments. Each registry had a different definition of cancer, with some likely including patients for whom a cancer diagnosis in the past may no longer be relevant. Can we therefore define the characteristics of patients with cancer who have increased risks of worse outcomes following SARS-Cov-2 infection?

### What characteristics of patients with cancer predispose to increased risk from SARS-Cov-2?

Common risk factors for poorer outcomes following SARS-Cov-2 infection in patients with cancer include age, male sex, a history of smoking, increasing number of comorbidities, prior steroid use, and performance status. Cardiovascular comorbidities are also associated with poorer outcomes. These risk factors are similar to that of the general population who acquire COVID-19.^33,34^

### What cancer-specific characteristics increase the risk of mortality from SARS-Cov-2?

#### Tumour type

Haematological cancers have been associated with worse outcomes following SARS-Cov-2 infection.^16,23^ Focusing on solid tumours, thoracic cancers were initially identified as being associated with worse outcomes. The TERAVOLT study suggested a higher mortality rate for thoracic malignancies; however, this was when compared with contemporaneous published studies as the study did not directly compare with other solid cancer types.^20^ Results from the recently presented GCO-002 CACOVID-19 at the ESMO Congress 2020 supports the findings of worse outcomes for patients with thoracic cancers.^35^ However, other multinational and national registries have not detected an association between thoracic malignancies and increased risk of death, as seen in Table 1. A study in a New York hospital system and a multicentre study in China^26,29^ demonstrated that patients with lung cancer had a higher risk of adverse outcomes when compared with other cancer types (55% vs 28%, and 18.8% vs 11.1%, respectively). Potential reasons for patients with thoracic tumours having worse outcomes may include age, pre-existing lung comorbidities, a history of smoking and potentially lower respiratory reserve due to lung cancer or previous interventions including surgery and radiotherapy. A multivariate analysis in the TERAVOLT study demonstrated that chronic obstructive pulmonary disease (COPD), hypertension, male sex, older age and a history of smoking were risk factors for worse prognosis.^20^

#### Tumour stage or status

The OnCovid study did not demonstrate an association between advanced cancer stage and outcomes. However, active cancer compared with those in remission or with no measurable disease was an independent risk factor for death (HR 1.81, 95% CI 1.35–2.44; *P* < 0.0001).^19^ This association with disease trajectory was also seen in the CCC-19 cohort. In the CCC-19 study,^14^ disease which was stable or responding to treatment was associated with increased risk of mortality compared with patients who were in remission or had no evidence of disease with an OR 1.79 (95% CI 1.09–2.95). Those with progressive disease were found to have an even higher increased OR at 5.20 (95% CI 2.77–9.77). In the updated analysis of 3887 patients from the CCC-19 registry presented at the ESMO 2020 congress, progressive cancer was shown again to be associated with increased risks for mortality following SARS-Cov-2 infection with adjusted odds ratio (aOR) for 30-day all-cause mortality of 2.9 (95% CI 2.1–4.0).^15^

A multicentre study in China^26^ comprising of 105 hospitalised patients showed that metastatic disease (stage IV) was associated with higher risks of death (OR 5.58, 95% CI 1.71–18.23; *P* = 0.01), ICU admission (OR 6.59, 95% CI 2.32–18.72; *P* < 0.01), and use of invasive mechanical ventilation (OR 55.42, 95% CI 13.21–232.47; *P* < 0.01).

Taken together, the data from larger studies indicate that the trajectory of a patient’s cancer and how it is responding to treatment may affect outcomes following SARS-Cov-2 more so than stage. The mechanisms by which advancing disease or stage affect SARS-Cov-2 outcomes are yet to be demonstrated. Patient fitness which can be negatively impacted due to progressive or metastatic cancer may be a risk factor, and this is in keeping with poorer PS being a risk factor for adverse outcomes (Table 1). Advanced or advancing disease is also associated with chronic inflammation and T-cell dysfunction and exhaustion which may also explain the poorer outcomes for these patients.^36^

#### Systemic anti-cancer therapy (SACT)

It was initially assumed that SACT could lead to worse outcomes following SARS-Cov-2 infection, which was further supported by early data from a small cohort of patients during the first wave.^5^ Chemotherapy generally has an immunosuppressive effect. Patterns of SACT prescribing, especially chemotherapy, were altered globally to try to reduce this hypothetical risk to patients. The safety and feasibility of delivering SACT remains an ongoing concern to patients and oncologists.

SACT comprises many different treatment modalities including chemotherapy, immunotherapy, targeted therapies, and hormone therapies. There are many different drugs and drug combinations, each with different mechanisms of action. Many studies have grouped treatments to analyse the risks for poorer COVID-19 outcomes following SACT. It is unlikely that associations between specific SACT regimens and COVID-19 outcomes will be seen without more targeted studies. Further, it is unlikely that patients receiving SACT during the pandemic reflect the population of cancer patients who would normally have been considered for SACT before the pandemic. These caveats must be borne in mind when interpreting registry data which, to date, is almost exclusively based on data obtained during the first wave.

Initial data from these registries did not demonstrate an association between SACT and mortality (Table 1). Recent evidence has been more conflicting with regards to the risks between SACT and mortality following SARS-Cov-2 infection. Changes in patient selection for SACT and changes to more “COVID-19 safe” regimens may also further confound matters. In the second OnCovid study,^19^ receipt of SACT was associated with better outcomes (HR 0.71, 95% CI 0.53–0.95; *P* = 0.019). These findings may be explained by patient selection and is likely to be associative rather than causative as the cohort of patients receiving SACT in that study were younger, less comorbid, and proportionally more female.

In the ISARIC study, active cancer treatment was associated with increased mortality in those aged 50 and above with the risk of mortality increasing with age. However, there was no further information available on the types of malignancies nor information on the active cancer treatments received.^18^ The second UKCCMP analysis found that patients with haematological malignancies who received SACT had an increased risk of mortality. However, no association was found in patients with other tumour types following SACT.^16^

The CCC-19 consortium presented data at the ESMO 2020 congress exploring the temporal association between SACT and mortality. They categorised patients into those who received SACT < 2 weeks, 2–4 weeks, 1–3 months or 3–12 months before a diagnosis of COVID-19. Receipt of chemotherapy or chemotherapy and immunotherapy within 2 weeks of being diagnosed with SARS-Cov-2 was associated with increased mortality (Table 1).^11^ Further numbers of patients are needed to confirm this finding, especially as the lower limits of the confidence interval for both groups approached 1 and haematological patients comprised about a quarter of the cohort. Interestingly, in the updated TERAVOLT study presented at the ESMO 2020 congress, chemotherapy or no therapy was associated with poorer outcomes when compared with patients receiving immunotherapy, chemotherapy-immunotherapy or targeted therapies.^21^ Patients who were not receiving treatments appeared to have the worst outcomes. Performance status, stage of disease, smoking history, prior steroid use and age were found to have a larger effect on mortality. This indicates that patient selection could be the reason why patients who were not receiving treatment or patients on chemotherapy did worse when compared with other treatment modalities, as these patients with lung cancer are usually on later lines of anti-cancer treatment.

#### Radiotherapy

Radiotherapy services were similarly adjusted during the first wave of the global pandemic. It is likely that the risk posed by radiotherapy is primarily due to the in-person contact required, sometimes daily over several weeks, rather than radiotherapy itself. No study has identified an association between recent radiotherapy and mortality, although the number of patients on whom conclusions can be drawn remain surprisingly small. For example, in TERAVOLT, only 13 thoracic cancer patients of the 147 on active therapy were receiving radiotherapy (stand-alone or in combination with SACT).^20^ In CCC-19 and UKCCMP, 12 and 76 patients respectively had recently had radiotherapy.^14,22^ Given the likely significant rise in radiotherapy given delays in diagnosis, pausing of SACT and reduction in surgical capacity during the first wave, an evidence-based strategy will be vital to guide clinical teams and their patients. In the UK, the National Cancer Research Institute’s Clinical and Translational Radiotherapy (NCRI CTRad) has launched COVID RT to study the impact of SARS-Cov-2 on radiotherapy and outcomes.^37^

## Conclusions

The SARS-Cov-2 pandemic has had, and continues to have, an impact on patients with cancer. Personalised, evidenced-based oncological care is important to safeguard the wellbeing of patients with cancer and to ensure that anti-cancer treatment can continue as safely as possible to avoid compromising overall outcomes. In this review, we have discussed the findings from key large national and international studies which were established at a short notice.

Overall, these studies suggest that patients with cancer are at an increased risk of mortality following SARS-Cov-2 infection compared with patients without cancer. Clinical factors such as age, male sex, number of comorbidities, cardiopulmonary comorbidities, smoking history, and performance status have all been associated with worse outcomes in patients with cancer who acquire SARS-Cov-2. Cancer-specific features that have been identified as being associated with worse outcomes include tumour stage and disease progression, with some studies identifying thoracic cancers as being associated with increased risk compared with other solid tumours. The majority of the studies discussed did not demonstrate an association between SACT and worse outcomes. However, recent data suggest that timing of treatment in relation to acquisition of COVID-19 may be significant with receipt of chemotherapy and chemotherapy-immunotherapy within 2 weeks associated with worse outcomes. This may be useful in advising patients about shielding in a treatment-specific manner. Larger studies with more patient numbers are needed to confirm these findings especially with regards to timing of SACT and patient outcomes.

The cancer community responded rapidly to develop these multicentre registries and they are highly heterogeneous in their definitions. Consequently, comparisons are difficult, and they must be interpreted with the cautions outlined above. Nonetheless, they have contributed immensely to our understanding of the risks to patients with cancer following SARS-Cov-2 infection during the first peak of the pandemic. Ongoing recruitment to such registries will help our evolving understanding regarding the interaction of SARS-Cov-2 and cancer, including timing of SACT, safety of individual SACT regimens and SACT in specific tumour types.

## Data Availability

Not applicable.
